# MATLAB-based algorithm to estimate depths of isolated thin dike-like sources using higher-order horizontal derivatives of magnetic anomalies

**DOI:** 10.1186/s40064-016-3030-7

**Published:** 2016-08-22

**Authors:** Yunus Levent Ekinci

**Affiliations:** 1 Department of Archaeology, Faculty of Arts and Sciences, Bitlis Eren University, 13000 Bitlis, Turkey; 2 Career Research and Application Center, Bitlis Eren University, 13000 Bitlis, Turkey

**Keywords:** Depth determination, Magnetic anomalies, Higher-order horizontal derivatives, Dike-like magnetized bodies, Open source code

## Abstract

This paper presents an easy-to-use open source computer algorithm (code) for estimating the depths of isolated single thin dike-like source bodies by using numerical second-, third-, and fourth-order horizontal derivatives computed from observed magnetic anomalies. The approach does not require a priori information and uses some filters of successive graticule spacings. The computed higher-order horizontal derivative datasets are used to solve nonlinear equations for depth determination. The solutions are independent from the magnetization and ambient field directions. The practical usability of the developed code, designed in MATLAB R2012b (MathWorks Inc.), was successfully examined using some synthetic simulations with and without noise. The algorithm was then used to estimate the depths of some ore bodies buried in different regions (USA, Sweden, and Canada). Real data tests clearly indicated that the obtained depths are in good agreement with those of previous studies and drilling information. Additionally, a state-of-the-art inversion scheme based on particle swarm optimization produced comparable results to those of the higher-order horizontal derivative analyses in both synthetic and real anomaly cases. Accordingly, the proposed code is verified to be useful in interpreting isolated single thin dike-like magnetized bodies and may be an alternative processing technique. The open source code can be easily modified and adapted to suit the benefits of other researchers.

**Electronic supplementary material:**

The online version of this article (doi:10.1186/s40064-016-3030-7) contains supplementary material, which is available to authorized users.

## Background

Geomagnetic surveys aim to investigate subsurface geology through the anomalies in the Earth’s magnetic field originating from magnetic minerals contained in subsurface rocks (Kearey et al. 2002). The estimation of model parameters of magnetic sources, such as location, depth, thickness, dip, size, shape, extension, and magnetic susceptibility, is extremely important in the interpretation stage. However, the well-known complex nature of the magnetic anomalies may complicate the interpretation. According to the Gauss theorem, if the potential field is known only on a bounding surface, there may be infinitely many equivalent causative source distributions inside the boundary that can produce the same anomaly characterization (Li and Oldenburg 1996). In some cases, remanent magnetization mostly produces noteworthy effects, which may lead to incorrect interpretations if overlooked (Telford et al. 1990). Data processing techniques noticeably assist in the interpretation of potential field anomalies and may also aid in geological implications and mapping (Blakely 1995). Hence, depending on the objectives of analyses and studies, many processing techniques have been reported for interpreting potential field anomalies. If potential field data quality permits, numerous analysing procedures can be conducted that facilitate in building a general understanding of the details of causative bodies (Ekinci and Yiğitbaş 2012, 2015; Balkaya et al. 2012; Ekinci et al. 2013, 2014).

Generally, model parameters of causative structures are frequently analysed and estimated using spectral methods, inversion and modelling techniques, graphical methods, and other numerical methods (Ekinci and Sarı 2008). To determine model parameters such as source depth, magnetic anomalies are commonly interpreted using some simple-shaped geometric source bodies such as point source, sheet, sphere, horizontal and vertical cylinders, and prism (Gay 1963, 1965; Mohan et al. 1982; Prakasa Rao et al. 1986; Rao and Babu 1991; Abdelrahman and Sharafeldin 1996). Further, some studies have reported a number of automatic methods, such as Werner (Werner 1953) and Euler (Thompson 1982; Reid et al. 1990) deconvolution methods, in which the depth estimation problem is transformed into the problem of determining a solution to a system of linear equations (Abdelrahman and Abo-Ezz 2001). One of the efficient numerical methods to estimate the depth at the top of a isolated dike-like body involves analysing the numerical second-, third-, and fourth-order horizontal derivative anomalies of magnetic data computed using some filters of successive graticule spacings (Abdelrahman and Abo-Ezz 2001). By considering many recent studies focused on determining the depths to the top of isolated thin dike-like magnetic sources (Bastani and Pedersen 2001; Abdelrahman and Essa 2005; Asfahani and Tlas 2007; Tlas and Asfahani 2011a, b; Cooper 2012, 2014, 2015), it was assumed that it might be favourable to develop a computer code to implement a depth-determination procedure for the benefit of scientific studies and investigations. The algorithm based on the use of higher-order horizontal derivative analyses is designed using MATLAB R2012b (Mathworks Inc.). To evaluate the efficiency of the developed code, synthetically produced magnetic anomalies with and without noise and some real magnetic anomalies from Arizona (USA), Kiirnunavaara (Sweden), and Ontario (Canada) were analysed. Applications clearly showed that the results obtained from the proposed code, particle swarm optimization (PSO), and previous studies are comparable.

## Methods

### Higher-order horizontal derivative analyses and depth determination

The general expression for a magnetic anomaly either in total, vertical, or horizontal fields of an arbitrary magnetized thin dike-like structure is given by (Gay 1963; Atchuta Rao et al. 1980; Sundararajan et al. 1985; Abdelrahman and Sharafeldin 1996; Asfahani and Tlas 2007; Tlas and Asfahani 2011a, b)

1 $$\begin{aligned} & {\text{T}}\left( {{\text{x}}_{\text{i}} ,{\text{xo}},{\text{A}},{\text{z}},\uptheta} \right) = {\text{A}}\frac{{{\text{z}}\,{ \cos }\,\uptheta + \left( {{\text{x}}_{\text{i}} - {\text{xo}}} \right)\sin \,\uptheta}}{{\left( {{\text{x}}_{\text{i}} - {\text{xo}}} \right)^{2} + {\text{z}}^{2} }} \\ & \quad {\text{i}} = 1,2,3,4,5, \ldots ,{\text{N}} \\ \end{aligned}$$

where A = K z, and z represents the depth to the top of the magnetized thin dike, K is the amplitude coefficient or effective intensity of magnetization, θ is the effective angle of magnetization or the index parameter, and x and xo represent the horizontal position coordinates on the profile and the exact origin of the anomaly, respectively. By using this formula, it is implicitly assumed that the source structure is perpendicular to the profile direction. To implement the depth estimation procedure, numerical values of the higher-order horizontal derivatives of magnetic data are required. Second-order horizontal derivatives are obtained by

2 $${\text{T}}_{2} \left( {{\text{x}}_{\text{i}} } \right) = \frac{{{\text{T}}\left( {{\text{x}}_{\text{i}} + 2{\text{s}}} \right) - 2{\text{T}}\left( {{\text{x}}_{\text{i}} } \right) + {\text{T}}\left( {{\text{x}}_{\text{i}} - 2{\text{s}}} \right)}}{{\left( {2{\text{s}}} \right)^{2} }}$$

where T_2_ represents the second-order horizontal derivative, T represents the magnetic data, x is the horizontal position coordinate, and s is the graticule spacing or numeric sample interval (i.e., 2, 3, 4, and 5). The nonlinear equation used for depth estimation is derived using Eq. (1) and is given by the following expression (see Abdelrahman and Abo-Ezz 2001 for detailed descriptions)

3 $${\text{z}} = \left. {\left[ {\frac{{{\text{F}}\left( {9{\text{s}}^{2} + {\text{z}}^{2} } \right)\left( {{\text{s}}^{2} + {\text{z}}^{2} } \right)\left[ {{\text{z}}^{3} - {\text{z}}\left( {4{\text{s}}^{2} + {\text{z}}^{2} } \right)} \right]}}{{\left( {4{\text{s}}^{2} + {\text{z}}^{2} } \right)\left[ {\left( {{\text{s}}^{2} + {\text{z}}^{2} } \right)\left( {\text{z}} \right) - \left( {9{\text{s}}^{2} + {\text{z}}^{2} } \right)\left( {\text{z}} \right)} \right]}}} \right.} \right]^{1/2}$$

where

4 $${\text{F}} = \frac{{{\text{T}}_{2} \left( {\text{s}} \right) + {\text{T}}_{2} \left( { - {\text{s}}} \right)}}{{{\text{T}}_{2} \left( 0 \right)}}$$

where T_2_ (0) represents the origin of the profile, which can be located practically by drawing a straight line joining the maximum and minimum values of the magnetic anomaly profile and locating the vertical axis by its intersection with the anomaly curve (Stanley 1977; Abdelrahman and Hassanein 2000; Abdelrahman and Abo-Ezz 2001; El-Araby 2003; Abdelrahman et al. 2012). In Eq. (3), the right- and left-hand terms involve the parameter z, which is the depth to the top of the dike-like magnetic source. This nonlinear equation is solved easily by using an iterative method (Press et al. 2007) in the form of

5 $${\text{z}}_{\text{u}} = {\text{f}}\left( {{\text{z}}_{\text{i}} } \right)$$

where z_u_ is the updated depth and z_i_ is the initial depth (close to zero; e.g., 1e-1). In each iteration, z_u_ is used as the initial estimate and the iteration terminates when the difference between z_u_ and z_i_ reaches a user-defined small value close to zero (e.g., 1e-5). By using the simple finite-difference approximation, the third-order horizontal derivatives of the magnetic data are obtained as follows

6 $${\text{T}}_{3} \left( {{\text{x}}_{\text{i}} } \right) = \frac{{{\text{T}}\left( {{\text{x}}_{\text{i}} + 3{\text{s}}} \right) - 3{\text{T}}\left( {{\text{x}}_{\text{i}} + {\text{s}}} \right) + 3{\text{T}}\left( {{\text{x}}_{\text{i}} - {\text{s}}} \right) - {\text{T}}\left( {{\text{x}}_{\text{i}} - 3{\text{s}}} \right)}}{{\left( {2{\text{s}}} \right)^{3} }}$$

and the nonlinear equation derived from Eq. (1) becomes

7 $${\text{z}} = \left. {\left[ {\left( {\frac{{3{\text{F}}\left( {4{\text{s}}^{2} + {\text{z}}^{2} } \right)\left( {16{\text{s}}^{2} + {\text{z}}^{2} } \right)}}{{4\left( {9{\text{s}}^{2} + {\text{z}}^{2} } \right)}}} \right.} \right.\left. {\left[ {\frac{{\left( {{\text{s}}^{2} + {\text{z}}^{2} } \right) - \left( {9{\text{s}}^{2} + {\text{z}}^{2} } \right)}}{{\left( {4{\text{s}}^{2} + {\text{z}}^{2} } \right) - \left( {16{\text{s}}^{2} + {\text{z}}^{2} } \right)}}} \right]} \right) - {\text{s}}^{2} } \right)^{1/2}$$

where

8 $${\text{F}} = \frac{{{\text{T}}_{3} \left( {\text{s}} \right) + {\text{T}}_{3} \left( { - {\text{s}}} \right)}}{{{\text{T}}_{3} \left( 0 \right)}}$$

determines the depth to the top of the magnetized body by using third-order horizontal derivatives (Abdelrahman and Abo-Ezz 2001). Similarly, by using the finite-difference approximation, numerical values of fourth-order horizontal derivatives are obtained by

9 $${\text{T}}_{4} ({\text{x}}_{\text{i}} ) = \frac{{{\text{T}}({\text{x}}_{\text{i}} + 4{\text{s}}) - 4{\text{T}}({\text{x}}_{\text{i}} + 2{\text{s}}) + 6{\text{T}}({\text{x}}_{\text{i}} ) - 4{\text{T}}({\text{x}}_{\text{i}} - 2{\text{s}}) + {\text{T}}({\text{x}}_{\text{i}} - 4{\text{s}})}}{{\left( {2{\text{s}}} \right)^{4} }}$$

and the nonlinear depth equation derived from Eq. (1) is given as (Abdelrahman and Abo-Ezz 2001)

10 $${\text{z}} = \left[ {\frac{\text{FA}}{\text{B}}} \right]^{1/2}$$

where

11 $${\text{F}} = \frac{{{\text{T}}_{4} \left( {\text{s}} \right) + {\text{T}}_{4} \left( { - {\text{s}}} \right)}}{{{\text{T}}_{4} \left( 0 \right)}}$$

12 $${\text{A}} = \frac{{\left( {{\text{z}}^{2} \left( {4{\text{s}}^{2} + {\text{z}}^{2} } \right)\left( {\text{z}} \right) - 4{\text{z}}^{2} \left( {16{\text{s}}^{2} + {\text{z}}^{2} } \right)\left( {\text{z}} \right) + 3{\text{z}}\left( {16{\text{s}}^{2} + {\text{z}}^{2} } \right)\left( {4{\text{s}}^{2} + {\text{z}}^{2} } \right)} \right)}}{{\left( {16{\text{s}}^{2} + {\text{z}}^{2} } \right)\left( {4{\text{s}}^{2} + {\text{z}}^{2} } \right)}}$$

13 $${\text{B}} = \frac{{2{\text{z}}}}{{\left( {{\text{s}}^{2} + {\text{z}}^{2} } \right)}} - \frac{{3{\text{z}}}}{{\left( {9{\text{s}}^{2} + {\text{z}}^{2} } \right)}} + \frac{\text{z}}{{\left( {25{\text{s}}^{2} + {\text{z}}^{2} } \right)}}$$

Again, Eq. (5) is used to determine the global minimum.

### Inversion through PSO

It is known that global optimization algorithms as samplers are more suitable for achieving sampling during optimization. The main advantage of these algorithms is their ability to escape from local minima by performing a stochastic search within the model space (Balkaya 2013; Ekinci et al. 2016). Moreover, to determine the global minimum, they do not need a well-constructed initial estimate as they provide a robust and versatile search process. PSO (Kennedy and Eberhart 1995), a global optimization method, is one of the popular naturally inspired metaheuristic algorithms based on the behaviour of bird flocks and fish schools searching for food (Pallero et al. 2015). In brief, a user-defined objective function is optimized through a swarm of particles, searching the space of model parameters, whose responses are similar to the observed data. This stochastic population-based search algorithm is initialized by assigning a population of particles (a group of model parameters) with random positions (*x*) and velocities (*v*) in the search space (Göktürkler and Balkaya 2012). During inversion, position and velocity of each particle are updated using the following equations (Kennedy and Eberhart 1995; Shi and Eberhart 1998)

14 $$\begin{aligned} {\text{v}}_{{\text{i}}}^{{\text{k}} + 1} &= {\text{wv}}_{{\text{i}}}^{{\text{k}}} + {\text{c}}_{1} {\text{r}}_{1} \left( {{\text{p}}_{{\text{i}}}^{{\text{k}}} - {\text{x}}_{{\text{i}}}^{{\text{k}}} } \right) + {\text{c}}_{2} {\text{r}}_{2} \left( {{\text{g}}_{{\text{i}}}^{{\text{k}}} - {\text{x}}_{{\text{i}}}^{{\text{k}}} } \right) \\ \quad {\text{x}}_{{\text{i}}}^{{\text{k}} + 1} &= {\text{x}}_{{\text{i}}}^{{\text{k}}} + {\text{v}}_{{\text{i}}}^{{\text{k}}} \\ \end{aligned}$$

where ${\text{v}}_{{\text{i}}}^{{\text{k}}}$ is the velocity of the particle i at the kth iteration, ${\text{x}}_{{\text{i}}}^{{\text{k}}}$ is the current i model at kth iteration, *w* represents the value of the inertia weight (0 < *w* < 1), and c_1_ and c_2_ are the coefficients controlling the particle’s individual (i.e., best local value) and social behaviours (i.e., best global value), respectively. The symbols r_1_ and r_2_ are the random number generators (Press et al. 1994) drawn uniformly in the open interval [0, 1] (Srivastava and Agarval 2010). The iteration terminates after reaching the maximum number of iterations defined by the user or obtaining the desired objective function value (Shi and Eberhart 1998; Poli et al. 2007; Luke 2009; Salmon 2011; Peksen et al. 2011, 2014; Göktürkler and Balkaya 2012), which is defined as follows

15 $${\text{Err}} = \left[ {{\text{d}}_{{\text{obs}}} - {\text{d}}_{{\text{cal}}} } \right]^{{\text{T}}} \cdot \left[ {{\text{d}}_{{\text{obs}}} -{\text{d}}_{{\text{cal}}} } \right]/{\text{N}}$$

where the superscript T is the matrix transpose, N is the amount of data, and d_obs_ and d_cal_ represent the magnetic anomalies observed and calculated at T(x_i_). In this study, 10 independent runs were performed using 100 particles to obtain the optimum model parameters. Values 1, 2, and 2 were assigned for the inertia weight (w) and the cognitive and social scaling factors (c_1_ and c_2_), respectively (Kennedy and Eberhart 1995). The root-mean-square values were calculated by obtaining the square root of Eq. (15).

## The computer algorithm

The developed MATLAB-based code (HigherDerivatives.m) analyses magnetic profile datasets using higher-order horizontal derivatives. During code execution, the procedure first loads the two-column profile dataset, which is written in SURFER data (*.dat) file format (GOLDEN SOFTWARE). The first and second columns include the horizontal distances of the observation points over the profile and the corresponding magnetic readings, respectively. An input dialog box is then opened to store the sampling interval of the profile in meters and the maximal graticule spacing number. Although, the default value for the maximal graticule spacing number is five, the user can increase the number if the length of the dataset is suitable. Next, the designed algorithm computes the higher-order horizontal derivative data for the given graticule spacing values and displays them on the screen via a MATLAB figure. After computing the depths by utilizing the aforementioned nonlinear equations, the derived results are stored in a text file compatible with a Microsoft text document. The developed open source algorithm (Additional file 1: HigherDerivatives) and synthetic datasets (Additional file 2: Figure1data and Additional file 3: Figure2data) are given as Additional files in text format. The code and datasets must be copied into a MATLAB.m file and a worksheet in the SURFER program, respectively.

## Test studies

### Synthetic data examples

**Fig. 1:**
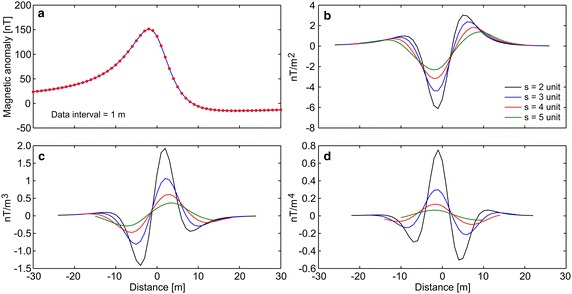
**a** Noise-free synthetic magnetic anomaly, **b**–**d** derived anomalies through the second-, third-, and fourth-order horizontal derivatives

**Fig. 2:**
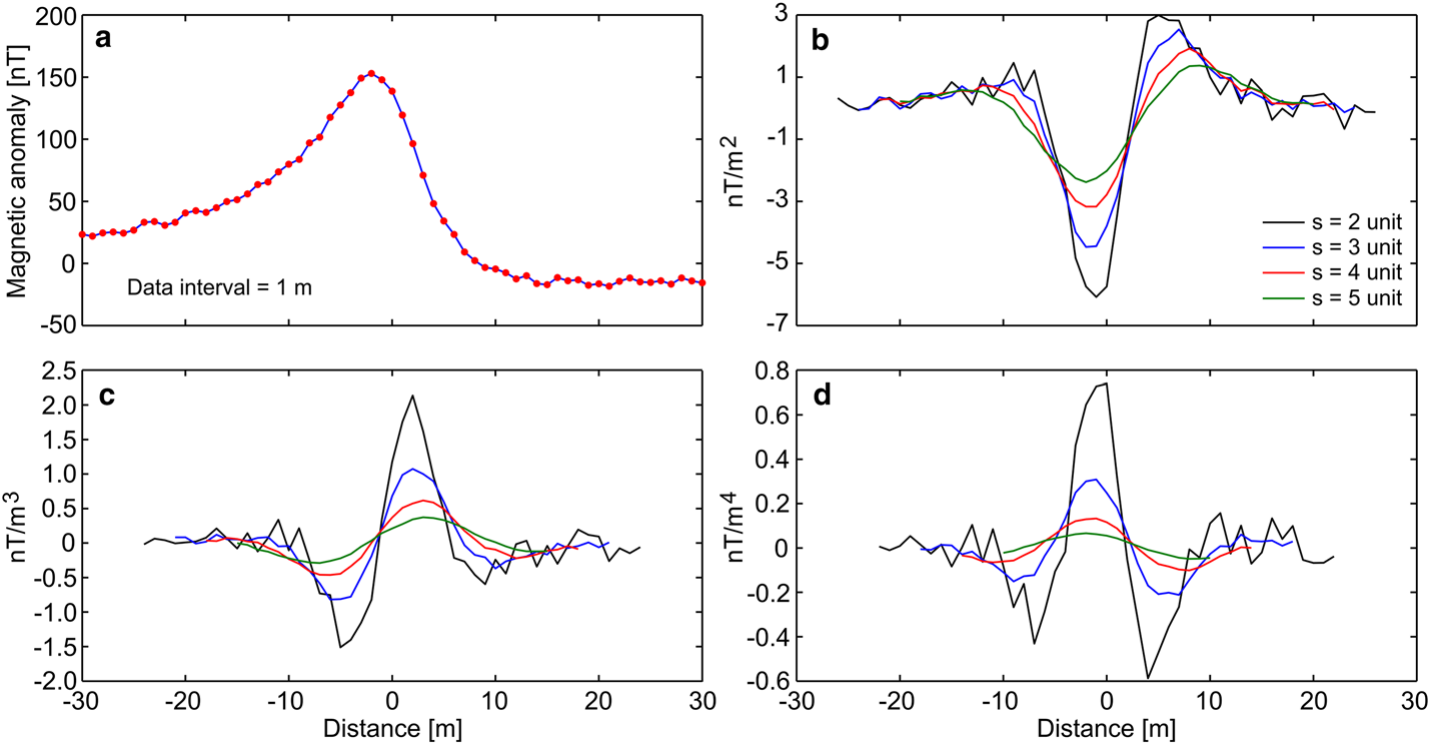
**a** Noisy synthetic magnetic anomaly, **b**–**d** derived anomalies through the second-, third-, and fourth-order horizontal derivatives

**Fig. 3:**
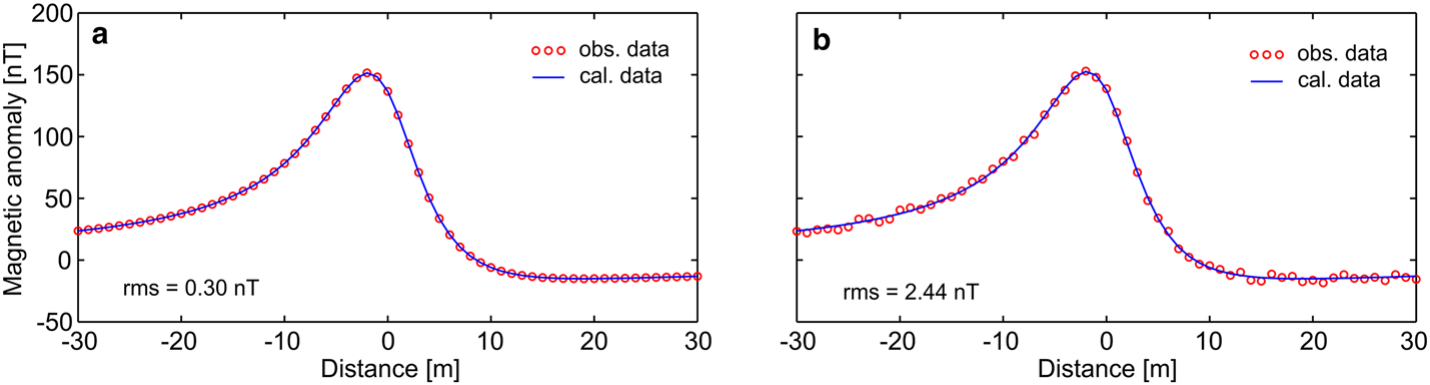
Synthetic magnetic datasets and the anomalies calculated by using the best-fitting model parameters obtained from PSO algorithm. **a** Noise-free example, **b** noisy example

First, the efficiency of the developed algorithm was tested by constructing some synthetic simulations with and without noise. Synthetic magnetic dataset was generated using Eq. (1). Figure 1a demonstrates the magnetic anomaly of the noise-free example with model parameters z = 6 m, A = 1000 nT m, θ = 35°, profile length = 60 m, and sampling interval = 1 m. Note that the exact origin is xo = 0. After obtaining second-, third-, and fourth-order horizontal derivatives using graticule spacings (s = 2, 3, 4, and 5 spacing units) (Fig. 1b–d), the nonlinear equations (Eqs. 3, 7, 10) were used to determine the depth to the top of the causative body. Table 1 lists the obtained results, which indicate that the depths were precisely estimated from higher-order horizontal derivatives for different graticule spacings. For the second example, the test data shown in Fig. 1a was contaminated by adding normally distributed zero-mean pseudo-random numbers with standard deviation of ±2 nT. Figure 2a shows the contaminated magnetic anomaly, and Fig. 2b–d demonstrate the anomalies derived by computing higher-order horizontal derivatives for different graticule spacings. The results clearly show that the average depths obtained from higher-order horizontal derivatives are very close to each other (Table 1). When considering the standard deviations of obtained depths, the third-order derivative produced an optimum result (5.94 ± 0.39 m). Although there is artificial noise in the magnetic dataset, the obtained average depth seemed to be very convincing. Additionally, the results obtained through higher-order horizontal derivative analyses were compared with those obtained using one of the state-of-the-art inversion techniques, namely PSO. As mentioned earlier, the inversion process was repeated 10 times by using different starting models, and the model having the minimum objective-function value (i.e., error) was considered the best-fitting model. Table 2 lists the search space parameters for PSO and the estimated depths. Figure 3 shows observed and calculated magnetic anomalies for both noise-free and noisy examples. According to the closeness of the results obtained using higher-order horizontal derivative analyses and the PSO technique in the synthetic examples, it was considered beneficial to compare the results obtained using the developed code with both PSO algorithm and previous studies to evaluate the effectiveness of the proposed code for real data cases.

**Table 1:** The depths obtained through higher-order horizontal derivative analyses for synthetic magnetic anomalies

Graticule spacing (s)	Second order derivative		Third order derivative		Fourth order derivative	
	Estimated depth (m)	Iteration number	Estimated depth (m)	Iteration number	Estimated depth (m)	Iteration number
*Synthetic model without noise*						
2	6.00	36	6.00	26	6.00	27
3	6.00	26	6.00	18	6.00	22
4	6.00	21	6.00	15	6.00	19
5	6.00	17	6.00	13	6.00	16
*Synthetic model with noise*						
2	5.14	31	6.05	26	4.82	24
3	6.30	27	5.44	17	6.44	23
4	5.79	20	6.39	16	5.81	18
5	6.15	18	5.87	13	6.17	17
Average depth (m)	5.84 ± 0.52		5.94 ± 0.39		5.81 ± 0.71

**Table 2:** Search space ranges and estimated parameters for synthetic magnetic anomalies

Model par.	True values	Search spaces		Estimated parameters	
		Min.	Max.	Noise-free data	Noisy data
z (m)	6	1	20	6.04	6.11
θ	−35	−90	90	−35.23	−36.23
A (nT m)	1000	10	10e3	1004.84	1015.97
rms (nT)				0.30	2.44
Run number at which the best solution obtained				2	1

### Real data examples

After the successful synthetic experiments, magnetic anomalies of a copper mine (Arizona, USA), an iron mine (Kiirunavaara, Sweden), and an olivine diabase dike (Ontario, Canada) were considered for investigating the effectiveness of the developed code on field datasets.

#### Pima copper mine, Arizona, USA

**Fig. 4:**
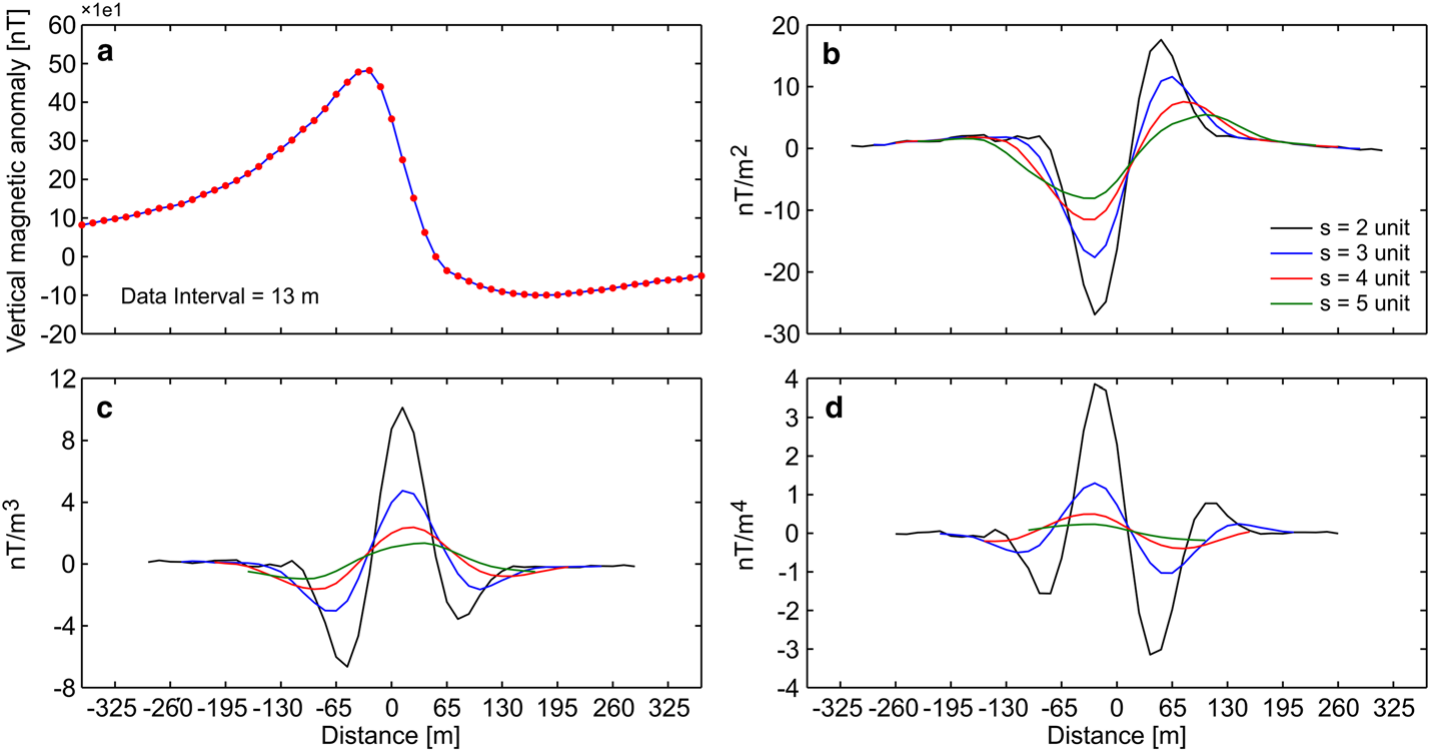
**a** Vertical component magnetic anomaly over Pima copper mine, Arizona, USA (adapted from Abdelrahman and Sharafeldin (1996), p 219), **b**–**d** derived anomalies through the second-, third-, and fourth-order horizontal derivatives

The first example includes a vertical component magnetic anomaly (Fig. 4a) obtained from the Pima copper mine, Arizona, USA (Gay 1963), which has been a major industry since the nineteenth century. The Pima mining district is one of the largest porphyry copper districts in USA. Mineralization related to Laramide igneous activity is known to occur in Paleozoic sedimentary rocks, Mesozoic sedimentary and volcanic sequences, and in Paleocene igneous rocks (Shafiqullah and Langlois 1978). The 728 m long vertical magnetic anomaly profile was digitized using a sampling interval of 13 m (Fig. 4a). The digitized magnetic anomaly was used to obtain the depth to the top of the ore body. Figure 4b–d show the anomalies derived from the use of different higher-order horizontal derivatives for different successive graticule spacings (s = 2, 3, 4, and 5). After obtaining the horizontal derivative anomalies, Eqs. 3, 7, and 10 were applied to compute the depth to the top of the copper ore dike. Table 3 shows the results: the average depths obtained from second-, third-, and fourth-order horizontal derivatives do not differ from each other significantly. The one with the lowest standard deviation yielded the optimal approximation. The depth to the top of the ore body computed using the developed algorithm is 67.9 m. The depth of this dike structure was previously reported by several researchers through different algorithms, and was recorded as 69.8 m (Gay 1963), 66 m (Abdelrahman and Sharafeldin 1996), 71.5 m (Asfahani and Tlas 2007), 71.25 m (Tlas and Asfahani 2011a), and 60 m (Abdelrahman and Essa 2015). Thus, the depth obtained using the developed code is very close to those of previous studies. Additionally, using the search space values, shown in Table 4, PSO algorithm produced a solution of 68.3 m (Fig. 7a), which matches well with the depth obtained using the developed code. Notably, the actual depth of the top of this thin dike body obtained by drilling is approximately 64 m (Gay 1963).

**Table 3:** The depths obtained through higher-order horizontal derivative analyses for Arizona (USA) vertical magnetic anomaly

Graticule spacing (s)	Second order derivative		Third order derivative		Fourth order derivative	
	Estimated depth (m)	Iteration number	Estimated depth (m)	Iteration number	Estimated depth (m)	Iteration number
2	64.12	30	58.30	20	60.54	23
3	65.20	22	56.26	14	61.62	19
4	69.39	19	61.43	13	68.89	17
5	72.78	16	68.19	12	74.35	16
Average depth (m)	67.87 ± 3.99		61.05 ± 5.22		66.35 ± 6.5

**Table 4:** Search space ranges and estimated parameters for Arizona (USA) vertical magnetic anomaly

Model par.	Search spaces		Estimated parameters
	Min.	Max.	Arizona data
z (m)	1	200	68.29
θ	−90	90	−50.76
A (nT m)	10	10e4	39267.31
rms (nT)			10.88
Run number at which the best solution obtained			2

#### Kiirunavaara iron mine, Sweden

**Fig. 5:**
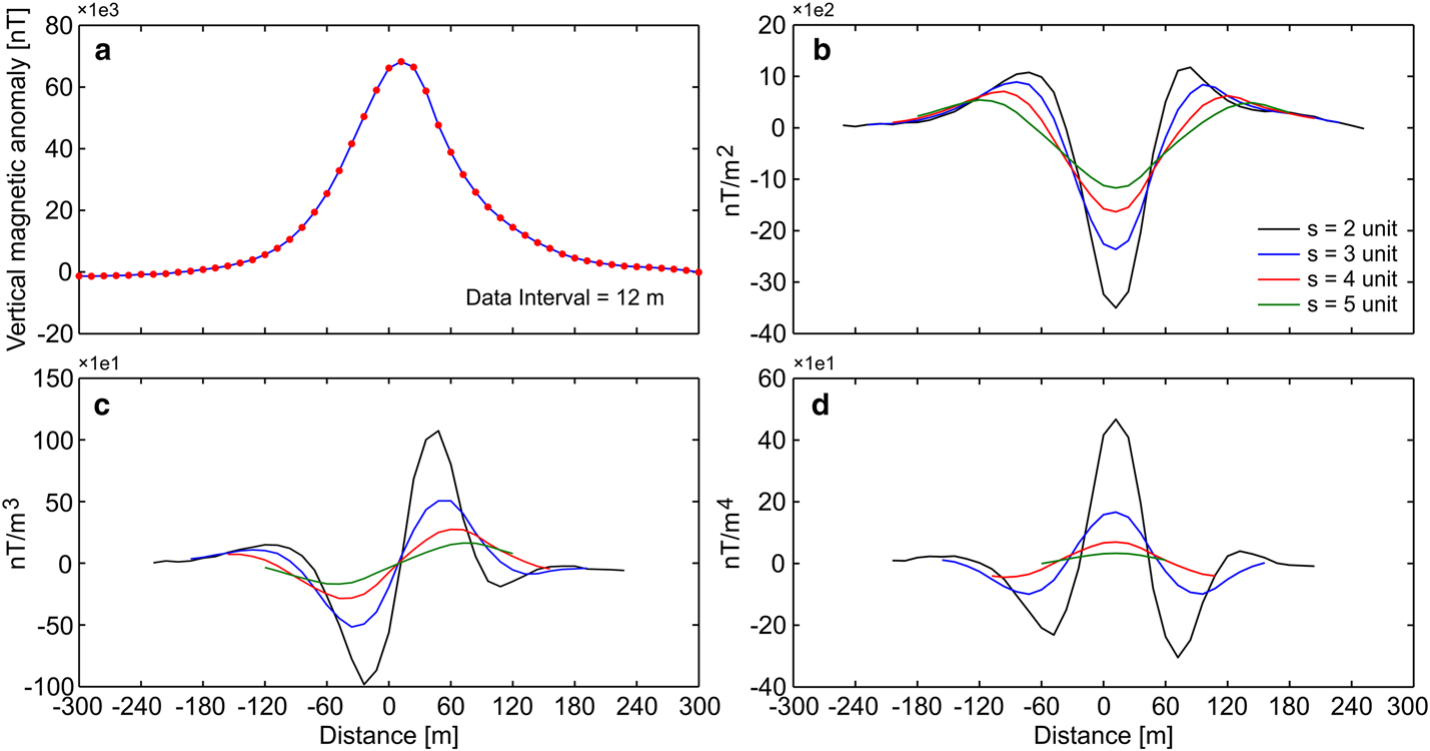
**a ** Vertical component magnetic anomaly over Kiirunavaara iron mine, Sweden (adapted from Sundararajan et al. (1985), p 564), **b**–**d** derived anomalies through the second-, third-, and fourth-order horizontal derivatives

The second field example is the vertical component of the magnetic anomaly observed at Kiirunavaara iron mine (northern Sweden), which is the largest of the apatite iron ores in Sweden. The Kiirunavaara group or Kiruna porphyries host economically important iron oxide-apatite deposits in the Kiruna and Malmberget areas (Lynch and Jönberger 2014). The vertical component magnetic anomaly used in this study is due to a vein of approximately 20 % magnetite (Grant and West 1965). The 600 m long vertical component magnetic anomaly was digitized with a sampling interval of 12 m (Fig. 5a). Figure 5b–d illustrate the anomalies obtained from higher-order horizontal derivatives for different graticule spacings. Table 5 lists the depths at the top of the ore body computed through Eqs. 3, 7, and 10. The results clearly show that the depths obtained by the use of second- and fourth-order horizontal derivatives are very close to each other, whereas the depth computed through the third-order horizontal derivative differs significantly. This may be due to the regional background, as suggested by Abdelrahman and Abo-Ezz (2001). The lowest standard deviation for the depths was obtained from the second-order horizontal derivatives and the average depth obtained is 65.4 m, which is close to the results of other studies: 59 m by Sundararajan et al. (1985) and 62–63 m by Grant and West (1965). Table 6 lists the search ranges used and the parameters obtained from PSO inversion. The PSO algorithm yielded a depth of 56.1 m (Table 6; Fig. 7b), which moderately supports the results of the higher-order horizontal derivative analyses.

**Table 5:** The depths obtained through higher-order horizontal derivative analyses for Kiirunavaara (Sweden) vertical magnetic anomaly

Graticule spacing (s)	Second order derivative		Third order derivative		Fourth order derivative	
	Estimated depth (m)	Iteration number	Estimated depth (m)	Iteration number	Estimated depth (m)	Iteration number
2	69.39	35	28.31	12	71.07	27
3	66.46	24	30.95	11	66.82	21
4	63.07	19	47.55	12	63.86	17
5	62.59	15	49.66	11	63.51	15
Average depth (m)	65.38 ± 3.18		39.12 ± 11.04		66.32 ± 3.5

**Table 6:** Search space ranges and estimated parameters for Kiirunavaara (Sweden) vertical magnetic anomaly

Model par.	Search spaces		Estimated parameters
	Min.	Max.	Kiirunavaara data
z (m)	1	200	56.09
θ	−90	90	10.39
A (nT m)	10	10e6	3713125.65
rms (nT)			2970.68
Run number at which the best solution obtained			10

#### Diabase dike, Pishabo Lake, Ontario, Canada

**Fig. 6:**
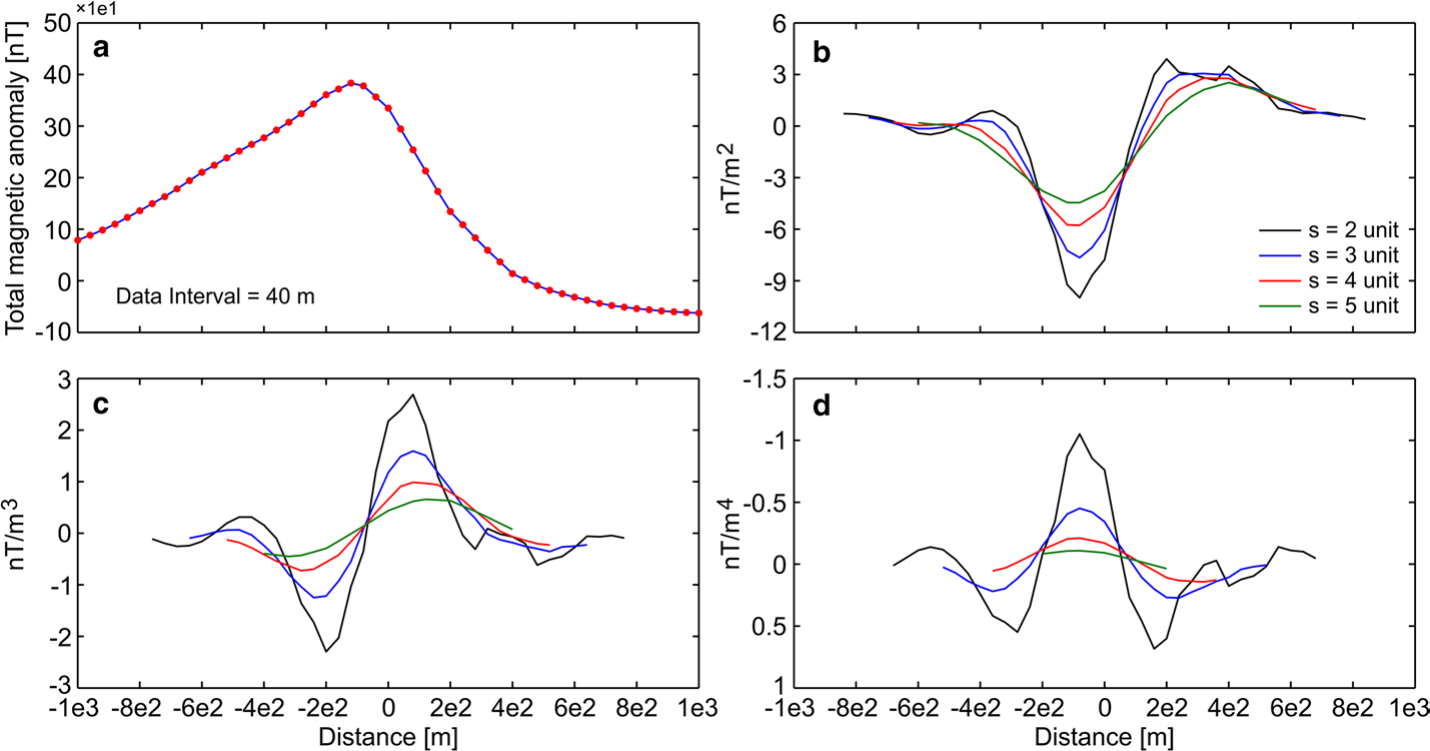
**a ** Total field magnetic anomaly over Ontario diabase dike, Canada (adapted from Abdelrahman et al. (2012), p 187), **b–d** derived anomalies through the second-, third-, and fourth-order horizontal derivatives

**Fig. 7:**
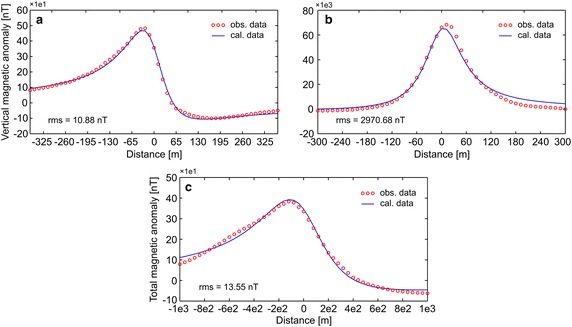
Observed datasets and the anomalies calculated by using the best-fitting model parameters obtained from PSO algorithm. **a** Pima copper mine, Arizona, USA example, **b** Kiirunavaara iron mine, Sweden example, **c** Ontario diabase dike, Canada example

The third example is a total field magnetic anomaly observed above an outcropping of a gabbroic olivine diabase dike, which intersects the northwestern arm of Pishabo Lake, Ontario, Canada (McGrath and Hood 1970). The airborne total field magnetic data have been collected with a flight elevation of approximately 304 m (McGrath and Hood 1970). The dike having a width of approximately 220 m (Abdelrahman et al. 2007) has been described as being composed of plagioclase, augite, biotite, apatite, olivine, and large patches of magnetite (El-Araby 2003). The other geological units in the study area are the granite gneiss and greywacke (McGrath and Hood 1970). A sampling interval of 40 m was used to digitize the 2000 m long total field magnetic anomaly. The digitized magnetic anomaly (Fig. 6a) was subjected to depth determination analyses. The anomalies obtained using higher-order horizontal derivatives for different graticule spacings are shown in Fig. 6b–d. Furthermore, Table 7 lists the computed depths and indicates that the average depth obtained from the second-order horizontal derivatives has the lowest standard deviation value. The obtained depth from the second-order horizontal derivatives is 319.5 m, which is in agreement with the flight height. In addition, the results of previous studies show close similarities: 294 m by El-Araby (2003), 317 m by Abdelrahman et al. (2007), 318.9 m by Abdelrahman et al. (2009), and 320 m by Abdelrahman et al. (2012). Moreover, the depth of 322.6 m obtained using PSO algorithm (see the details in Table 8; Fig. 7c) is very close to the depth obtained using the proposed code.

**Table 7:** The depths obtained through higher-order horizontal derivative analyses for Ontario (Canada) total magnetic anomaly

Graticule spacing (s)	Second order derivative		Third order derivative		Fourth order derivative	
	Estimated depth (m)	Iteration number	Estimated depth (m)	Iteration number	Estimated depth (m)	Iteration number
2	294.73	45	329.90	25	264.83	29
3	327.41	34	239.45	18	317.79	26
4	326.89	27	307.93	18	299.40	22
5	329.05	23	274.93	18	296.40	19
Average depth (m)	319.52 ± 16.55		288.05 ± 39.50		294.61 ± 21.99

**Table 8:** Search space ranges and estimated parameters for Ontario (Canada) total magnetic anomaly

Model par.	Search spaces		Estimated parameters
	Min.	Max.	Ontario data
z (m)	1	400	322.55
θ	−90	90	−37.81
A (nT m)	10	20e4	141600.27
rms (m)			13.55
Run number at which the best solution obtained			3

## Conclusions

An easy-to-use computer algorithm was developed in MATLAB to estimate the depths to the top of thin dike-like causative bodies by using higher-order horizontal derivatives of observed magnetic data. The proposed approach is based on the analyses of the numerical second-, third-, and fourth-order horizontal derivative anomalies obtained from the observed magnetic data by using some filters of successive graticule spacings. The nonlinear depth determination problem is rapidly solved in the code. The accuracy and effectiveness of the developed code were tested on synthetically produced magnetic datasets with and without noise. Additionally, the usability of the algorithm was evaluated by reanalysing some well-known magnetic anomalies from different parts of the world (USA, Sweden, and Canada). The results show that the outputs of the algorithm yielded satisfactory solutions, which are in good agreement with the actual, previously published, and PSO results. The main advantage of the proposed technique is that it does not need a priori information for determining the depth and can be easily used for short or long profile datasets having anomalies due to single thin dike-like sources. Further, the solutions are independent from the magnetization and ambient field directions, namely, inclination and declination angles. Consequently, the developed algorithm using higher-order horizontal derivative analyses was proved useful in interpreting magnetic anomalies observed over single isolated thin dike-like source bodies and may be an efficient tool in magnetic prospecting. Furthermore, one of the greatest benefits of the developed code is that it is an open source algorithm. Thus, it is easy to modify and adapt the algorithm to suit the benefits of the other researchers studying similar or special topics.

## Additional files

10.1186/s40064-016-3030-7 MATLAB code of the higher-order horizontal derivative analyses. The code given in text format must be copied into a MATLAB.m file.
10.1186/s40064-016-3030-7 Noise-free synthetic data shown in Fig. 1a. The dataset given in text format must be copied into a worksheet in the SURFER program.
10.1186/s40064-016-3030-7 Noisy synthetic data shown in Fig. 2a. The dataset given in text format must be copied into a worksheet in the SURFER program.
